# Diagnostic Dilemma in Appendiceal Mucormycosis: A Rare Case Report

**DOI:** 10.1155/2016/9531840

**Published:** 2016-08-18

**Authors:** Sali Priyanka Akhilesh, Yadav Kamal Sunder, Pande Prasad, George Mary Asha, Agarwal Mohan, Mehta Hitesh

**Affiliations:** ^1^Department of GI Surgery, Lilavati Hospital and Research Centre, A-791, Bandra Reclamation, Bandra West, Mumbai 400050, India; ^2^Department of Histopathology, Lilavati Hospital and Research Centre, A-791, Bandra Reclamation, Bandra West, Mumbai 400050, India; ^3^Department of Hematology, Lilavati Hospital and Research Centre, A-791, Bandra Reclamation, Bandra West, Mumbai 400050, India

## Abstract

Appendiceal mucormycosis is a rare life-threatening infection seen in immunocompromised patients. It is usually seen in chemotherapy induced neutropenia in patients with hematological malignancies. Clinically, the symptoms and signs may be masked due to ongoing corticosteroids. The condition may mimic bacterial appendicitis and the less serious condition, typhlitis. The disease demands prompt surgical debulking and aggressive antifungal treatment. However, surgery is delayed due to the poor performance status and severe neutropenia. This may lead to perforative peritonitis and further dissemination. The survival rates of such disease are dismal. Unfortunately, the diagnosis may be confirmed only on histological examination of the surgically excised tissue. Very few cases have been reported so far. We present here once such a fatal case of appendiceal mucormycosis in a 14-year-old boy who was immunosuppressed due to intensive induction therapy for Acute Myeloblastic Leukemia.

## 1. Introduction

Mucormycosis of appendix is a rare, usually fatal infection that is caused by subphylum Mucoromycotina, order Mucorales [1]. It is being increasingly encountered due to aggressive therapies used in malignancy and transplant recipients. Candidiasis and aspergillosis are the commonest culprits seen. Seen in immunosuppressed individuals predominantly in neutropenic stage of the underlying disease, If not suspected early, the disease is fatal. Aggressive surgical and medical therapy with intensive care is required to improve survival. It mimics the less aggressive typhlitis in its early stages that is usually managed conservatively. Very few cases have been reported. We present here a rare case of fatal appendiceal mucormycosis in a 14-year-boy who was on chemotherapy for Acute Myeloblastic Leukemia (AML).

## 2. Case Report

**Figure 1:**
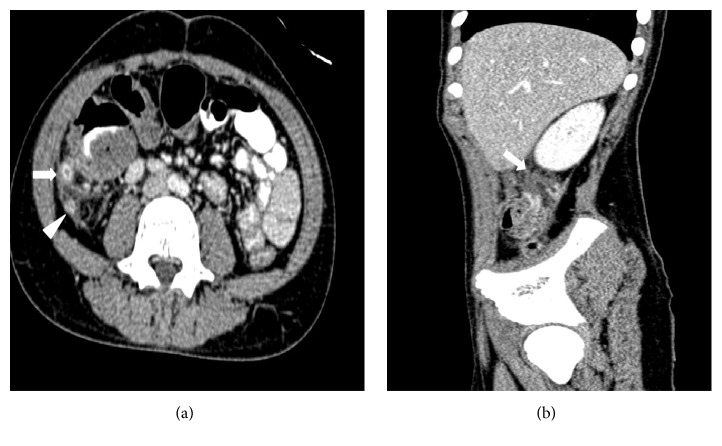
Contrast enhanced CT showing long, inflamed, and retrocaecal appendix with a distal kink. (a) Axial image showing inflamed (enhancing) appendix (proximal part-arrow and tip-arrow head). (b) Sagittal image showing the nonenhancing mid portion of the appendicular wall with kink (arrow) and no periappendicular collection.

**Figure 2:**
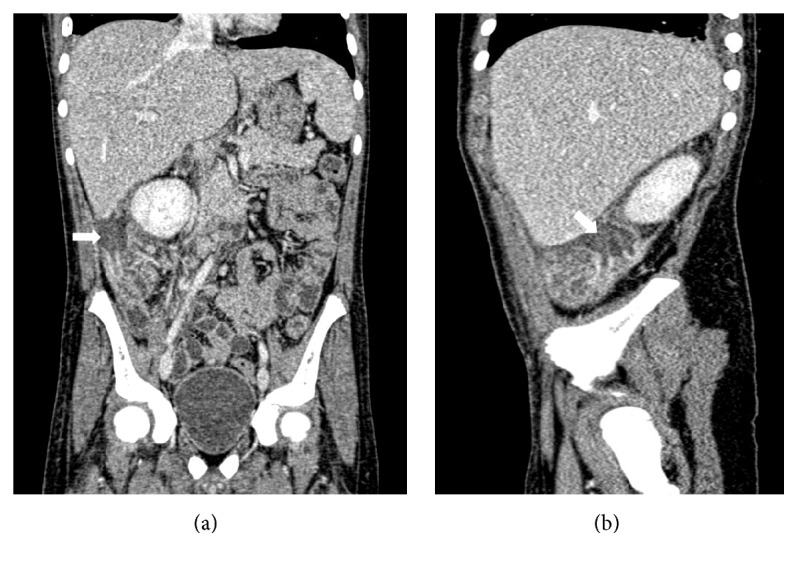
Contrast enhanced CT with coronal (a) and sagittal (b) images showing ruptured (arrow) appendix at the distal body with localized periappendicular collection.

**Figure 3:**
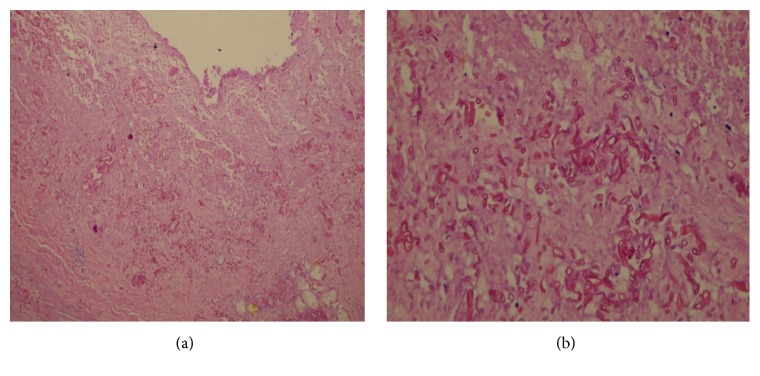
(a) H & E 100x showing necrotic appendicular wall with fungal colonies. (b) H & E 400x showing fungal colonies in the appendix.

**Figure 4:**
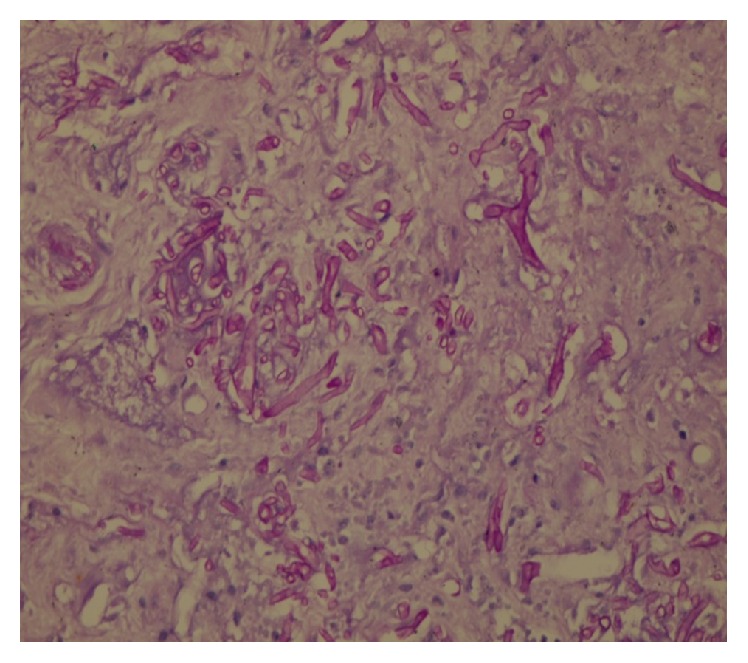
PAS stain confirming colonies of mucormycosis.

**Figure 5:**
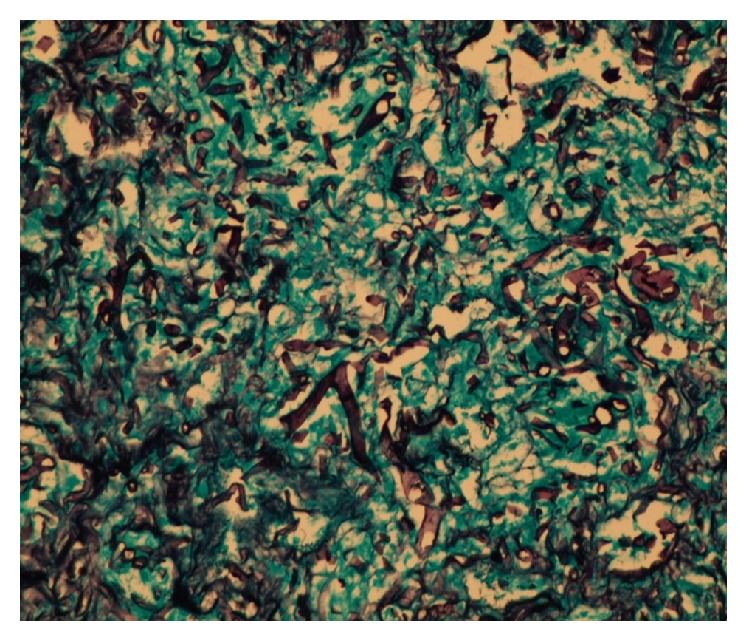
GMS stain confirming the broad aseptate hyphae of mucormycosis.

A 14-year-old boy recently diagnosed with AML since 15 days and on intensive induction chemotherapy with daunomycin and cytarabine, presented with right iliac fossa (RIF) pain and low grade fever for 2 days. On examination, he was mildly tachycardic and had tenderness in the right iliac fossa. No lump was felt. Abdominal ultrasonography showed acute appendicitis with minimal fluid collection. Findings were corroborated with Computed Tomography (CT) (Figures 1(a) and 1(b)). Due to neutropenia and thrombocytopenia (WBC: 240/cubic mm and platelets: 16,000/cubic mm), surgery was deferred and patient was managed conservatively with intravenous antibiotics and multiple packed cells and platelet transfusions. After 2 days, he developed tachycardia, with tenderness and guarding with a lump in RIF. CT now revealed an inflamed appendix with its tip and body replaced with loculated fluid collection, suggestive of perforated appendicitis (Figures 2(a) and 2(b)). The patient was taken up for emergency surgery after transfusion with platelets. Intraoperatively, a 200 mL of seropurulent collection was found in the periappendicular region. The appendix was inflamed, thick walled, and ruptured at the body. Laparoscopic appendectomy was performed. On postoperative day 2, the patient developed respiratory distress and generalized abdominal pain. He now had generalized guarding. He was put on assisted ventilation. A repeat CT scan showed minimal collection in RIF. Fluid culture from the abdominal collection was positive for* E. coli*. The histopathology showed gangrenous perforated appendicitis with angioinvasive mucormycosis. Extensive mucosal ulceration and necrosis were seen in appendicular wall with scattered small and large fungal colonies in mucosa, infiltrating the serosa and periappendicular tissue (Figures 3(a) and 3(b)). Broad aseptate hyphae with right angle branching was seen with Periodic acid–Schiff (Figure 4) and Gomori methenamine silver staining (Figure 5). The patient was started on sensitive antibiotics and liposomal amphotericin substituted by posaconazole. He continued to have fever, distended abdomen, respiratory distress even on assisted ventilation, and dropping urine output. Due to renal involvement secondary to fungal sepsis, hemodialysis was commenced. Despite rigorous intensive care, patient deteriorated and succumbed on the sixth postoperative day.

## 3. Discussion

Mucormycosis is seen in immunocompromised patients with malignancy, transplant recipients on immunosuppressive therapy, malnutrition, patients on deferoxamine, inflammatory bowel disease on corticosteroid therapy, and diabetic ketoacidosis with hematological malignancies being the commonest cause [2]. It usually affects the rhinocerebral and pulmonary systems [3]. Gastrointestinal system is rarely affected contributing to 3–7% of systemic mucormycosis with stomach being the commonest site [4]. Cytotoxic therapy given in malignancies disrupts protective gastrointestinal mucosal barrier, rendering it easily susceptible to fungal penetration. Appendiceal mucormycosis is rare. Infection, if not contained by aggressive therapy, rapidly progresses to hepatic and pulmonary spread by vascular invasion leading to thrombosis, infarction, hemorrhage, and dissemination [3].

Appendiceal mucormycosis presents similar to bacterial appendicitis with nausea, abdominal pain, diarrhea, and fever. Symptoms may be masked if on steroid therapy. Frequent cause of such symptoms is typhlitis that improves conservatively. Unresolving abdominal pain in neutropenic patients must raise suspicion for fungal infections.

Ultrasonography may show features of appendicitis mimicking bacterial appendicitis. Choi et al. have described CT findings highly suspicious of intestinal mucormycosis and have reported progressive worsening of bowel features. It initially begins with enhancing thickening of the bowel wall that in due course culminates with bowel wall thinning and necrosis [5]. If suspected, it may be confirmed with CT guided aspiration of the collection or biopsy of affected tissue [6].

Aggressive treatment is required early in course of disease, since it rapidly progresses to disseminated form with mortality rate of 70–100% [7]. Both medical and surgical therapy is required to improve survival. Liposomal amphotericin is the antifungal of choice. Liposomal variant is preferred due to better renal tolerance. Recent studies have also advocated use of newer agents like posaconazole. It has been reported to be more effective than drugs that are in routine use [8].

Surgical therapy is generally deferred due to neutropenic status, poor general condition, and disseminated disease. However, it may be inevitable to resort to aggressive surgical debulking of involved regions with antifungal therapy to increase survival [9, 10]. Postoperatively, bleeding from stoma site or mucormycosis of surgical wound has been reported [11].

Hyperbaric oxygen, nonsiderophore iron chelators, and cytokines have also been implicated [12]. However, more studies are necessary to validate their advantage [13].

Unfortunately, mucormycosis is rarely diagnosed preoperatively. Due to high mortality involved, high suspicion with aggressive therapy is imperative. Diagnosis can be confirmed only with biopsy of infected tissue. Aseptate, wide hyphae branching at right angles, is confirmatory [14, 15].

Larbcharoensub et al. [16] presented 3 similar cases. Despite surgical therapy, only one patient survived. Others progressed to fulminant dissemination.

Karanth et al. [11] reported an acute lymphoblastic leukemic patient on chemotherapy. Diagnosed with typhlitis, the patient was initially managed conservatively. Due to overwhelming peritonitis, she later underwent right hemicolectomy with end ileostomy that was complicated with fatal hemorrhage. Histopathology confirmed mucormycosis.

ter Borg et al. [17] reported fatal appendiceal mucormycosis with hepatic dissemination in a patient with chemotherapy induced granulocytopenia.

We highlight the rare occurrence of appendicular mucormycosis that progressed to perforated peritonitis. High mortality rate and poor treatment outcome justify the high index of suspicion.

## 4. Conclusion

We reemphasize the imperative urgency of disease suspicion and aggressive treatment. It must be suspected in patients with underlying conditions that hamper host defense mechanisms. Aggressive surgical debulking with antifungals may improve survival.
